# Adolescent access to health services in fragile and conflict-affected contexts: The case of the Gaza Strip

**DOI:** 10.1186/s13031-021-00379-0

**Published:** 2021-05-21

**Authors:** Bassam Abu Hamad, Nicola Jones, Ingrid Gercama

**Affiliations:** 1 Faculty of Public Health, Al-Quds University/ GAGE MENA Regional Director, 101, Tel El Hawa, Gaza, Palestine; 2 ODI Principal Research Fellow and Director of Gender and Adolescence: Global Evidence (GAGE), Overseas Development Institute, 203 Blackfriars Bridge Road, London, SE1 8NJ UK; 3 Applied Anthropologist, Anthrovision, Overhoeksplein 2, 1031 KS Amsterdam, the Netherlands

**Keywords:** Adolescence, Gaza, Sexual and reproductive health, Conflict, Gender norms

## Abstract

**Background:**

Enjoyment of physical and mental health is not only recognized as a human right but also as an integral part of development, as reflected in Sustainable Development Goal 3 to ensure healthy lives and promote well-being for all at all ages. The rapid physical and psychosocial changes that take place during adolescence have a strong influence on the rest of a persons life course, so investments in adolescent health services constitute a unique opportunity to reap inter-generational dividends. Yet the evidence base on adolescents access to health services, particularly in conflict-affected contexts, remains thin. This article explores adolescents access to health services in the Gaza Strip, and their experiences and perceptions of those services.

**Methods:**

The article draws on mixed methods research in the Gaza Strip conducted in 2016 and 2017 as part of the Gender and Adolescence: Global Evidence research programme. Data were collected from 240 male and female adolescents combining in-depth interviews, focus group discussions and a tablet-based survey. This study also draws on a participatory action pilot project engaging 12 boys and 23 adolescent girls aged 1519years old.

**Results:**

The findings underscore that gender normsespecially those pertaining to adolescent girls sexual purityshape adolescent health in multiple ways. Girls face increasing restrictions on their mobility, leaving them with limited opportunities for leisure or exercise, socializing with peers or seeking health services and information. Adolescent boys in Gaza do not face the same restrictions, but given the multiple political, economic and familial stressors, they are at high risk of substance abuse including smoking and involvement in peer violence. Moreover, our findings suggest that a range of socioeconomic, cultural and structural barriers prevent adolescents in Gaza from accessing quality and appropriate health care. Study participants cited the main challenges being an absence of preventive adolescent health initiatives and limited information on sexual and reproductive health, as well as drug shortages, high treatment costs, and inappropriate interactions with service providers.

**Conclusions:**

The article highlights the importance of designing and implementing conflict-sensitive and age- and gender-appropriate adolescent services and information and promoting preventive services targeted at adolescents.

## Background

Enjoyment of the highest attainable standard of physical and mental health is recognized as a human right [1] and as an integral part of development, as reflected in Sustainable Development Goal (SDG) 3, which aims to ensure healthy lives and promote well-being for all at all ages. Given that the rapid physical, psychosocial and behavioural changes that take place during adolescence influence the rest of a persons life course [2], investments in adolescent health constitute a unique opportunity for a triple dividend of health benefitsduring adolescence, during adulthood, and for subsequent generations [3, 4]. However, due to the protracted nature of conflict and refugee crises, millions of young people experience adolescence in politically turbulent contexts, with limited access to key services. Research on the health status of adolescent girls and boys in conflict-affected contexts, and their experiences and perceptions of health services, is particularly limited.

This article seeks to contribute to the evidence base by exploring the health vulnerabilities facing adolescent girls and boys drawing on mixed-methods research undertaken in the Gaza Strip in 2016 and 2017. It focuses on three areas: (1) the extent to which the health and health care-seeking experiences of adolescents are shaped by gender relations and norms; (2) the types of gender- and age-friendly health services and information available to adolescents and their families; and (3) adolescents perceptions of the quality of health services. The article begins with a brief overview of the evidence on age- and gender-responsive health services in fragile and conflict-affected contexts, and an overview of the Gazan context. It then describes the research methods used, before presenting the results of the research, focusing on the age-, gender- and context-specific dimensions of adolescents health experiences and service uptake. The concluding section discusses implications of the research findings for policy and programming in fragile and conflict-affected contexts.

### Adolescent-friendly health services

The World Health Organizations adolescent-friendly health services framework [5] highlights the importance of providing health services that meet minimum quality standards, tackle adolescents heterogenous health vulnerabilities, are non-judgmental and accessible, and include adolescent perspectives in service planning, delivery and evaluation. Hawke et al. [6], drawing on findings from a systematic review, underscore that in the case of mental health, adolescent-friendly services need to be integrated, inclusive, confidential and safe. They should include age-appropriate information materials and communication and counselling skills, and have a core value of youth voice [1]. As Jennings et al. point out [7], however, the evidence base on adolescent health, and especially adolescent sexual and reproductive health, is a neglected area in humanitarian settings, and one that requires urgent attention. Their findings highlight the dearth of age- and gender-disaggregated data on health service provision, uptake and outcomes.

### The Gazan context

The Gaza Strip offers an important lens through which to explore adolescent access to and perceptions of health services, given that it is one of the worlds most protracted conflict contexts. The key socioeconomic and environmental determinants of health for the Gaza population have been negatively affected by the ongoing conflict, involving 14years of siege and economic collapse, which has increased health-related vulnerabilities, particularly among women and children [8]. However, compared to other countries at a similar level of economic development, the Palestinian populations overall health outcomes are relatively good, partly due to strong performance of basic public health and primary health care functions [9]. Gaza performs better than many countries in the Middle East region on key indicators: the infant mortality rate is low (at around 22 per 1000 live births) and immunization coverage is at 95% for most vaccines [10]. There is near-universal coverage of antenatal care: all Gazan women deliver in health facilities, and there has been a noticeable reduction in the fertility rate [11]. Moreover, health insurance coverage is widespread (more than 90% of households). This said, insurance coverage does not meet peoples needs; few medicines are covered by insurance (if available at all), and there are limited specialist services and long waiting lists for surgeries [12]. While people are ordinarily able to access basic health services, access becomes very challenging during renewed outbreaks of conflict [13], and access to advanced services outside Gaza (such as oncology, radiotherapy, advanced cardiac and neurosurgery) remains very limited [12].

Adolescents (aged 1019years) comprise 22% of Gazas population [10]. While school attendance is relatively high (98% for basic education and 80% for secondary education) [14], adolescent girls and boys have limited access to higher education and employment opportunities due to the conflict (an estimated 60% of adolescents in Gaza are officially unemployed) [15]. Girls from mid-adolescence onwards face a high risk of child marriage and early childbearing (28.6% of Gazan women of reproductive age were first married before 18years) [16] which has ramifications on psychosocial wellbeing [13]. Girls in Gaza face a double burden, as they are constrained not only by the occupation, political turbulence, and poverty, but also by restrictive cultural norms [13]. Boys face their own unique challenges including child labour and violence [17].

There is a dearth of information about the specific health status of adolescents as a distinct social group in Gaza, mainly because issues around adolescent health are usually subsumed within childrens or young peoples health. The leading causes of death in Gaza are somewhat different for adolescents compared with the general population namely, heart disease resulting from congenital anomalies and other medical conditions, accidents, congenital anomalies (other than heart disease), malnutrition, and infectious diseases [18]. According to a study by the United Nations Population Fund (UNFPA), 16% of youth had a health problem in the preceding 2 weeks, while 3% had at least one chronic disease (including disability), with higher rates among males (4.9%) compared to females (2.3%) [10]. The youth survey by the Palestinian Central Bureau of Statistics (PCBS) found that 85.9% of females and 90% of males aged 1529 believed themselves to be in either excellent or very good health [15]; the remainder judged their health status as average or poor.

In terms of sexual and reproductive health, the most recent PCBS survey found that of married girls aged 1519, 84% were not using contraception, compared to 62% of married women aged 2024 [15]. In Gaza, family planning is usually initiated late, with the first contraceptive use tending to start only after the fourth or fifth child and after having at least one boy, reflecting a culture of son preference [10]. Gaps in family planning services include limited access to information on family planning methods and weak counselling, which affect service uptake. Nearly 30% of girls in Gaza are pregnant before the age of 18 and about half become mothers before the age of 20 [16]. Child marriage endangers adolescent girls health as it exposes them to early childbearing, while few adolescent mothers are able to follow the recommended intervals of pregnancy spacing given strong family pressures and very limited agency and influence in household and spousal decision-making.

Although education on sexual and reproductive health, including menstruation, has been incorporated into the school curriculum, it is not clear how fully this is implemented [19]. In Gaza, sexual and reproductive health education remains controversial, circumscribed by political, economic, cultural and religious factors. Social taboos are major obstacles to informed discussions, particularly in relation to young people [20]. Many adolescent girls reported feeling unprepared for puberty and the physical and emotional changes it brings, with 28% stating that this phase had caused them problems [21]. For instance, 22% had no idea about menstruation; 40% were afraid (and 19% embarrassed) when they first experienced a period; and 43% taught themselves how to clean their bodies during a period [21].

Of those aged 1517, 40% had not heard about sexually transmitted infections (STIs) (other than human immunodeficiency virus -HIV) and 20% had not heard of HIV [19]. While STI prevalence remains relatively low in Gaza, the lack of sexual and reproductive health education is likely to lead to increases in future. It is therefore critical to take preventive action, ensuring that integrated health services include sexual and reproductive health and non-discriminatory counselling. Indeed, evidence suggests that gendered social norms, which hold that girls virginity is central to family honour, largely preclude girls and young womens access to care services other than those related to maternity [19].

Existing literature suggests that adolescents satisfaction levels with health care service providers is low. Several studies have highlighted that providers attitudes are problematic and act as a constraint to adolescent service access [10, 13], and that adolescents concerns are predominantly related to poor quality interaction, counselling and communication with service providers [22]. Because Gazas health care system is curative rather than preventive, and staff are mostly disease-oriented [22], adolescents usually only go to a clinic when they have acute manifestations such as high fever or infection; otherwise, they use home remedies such as drinking herbs or eating garlic. Reports indicate that people increasingly rely on home remedies and cultural rituals, due to economic hardship and lack of trust in health care providers [8].

## Research methods

This article draws on findings from a mixed method research study undertaken in the Gaza Strip in camp and non-camp settings in 2016 and 2017 as part of the multi-country Gender and Adolescence: Global Evidence (GAGE) longitudinal research program. It includes findings from a participatory pilot research study conducted in 2016 involving 35 adolescents (12 boys and 23 girls) aged 1519years who were part of an adolescent empowerment program implemented by the Culture and Free Thought Association (CFTA), a local civil society organisation operating in Khanyounis in the Gaza Strip. Subsequently, in 2017, we also surveyed a convenience sample of 107 adolescents (52 boys, 55 girls) aged 1119years (using an interactive, tablet-based QuickTapSurvey module), complemented by a range of individual and group qualitative research interviews involving 132 adolescents including girls married as children. This included 10 focus group discussions (FGDs) with 39 boys and 58 adolescent girls aged 1019years old as well as 35 in-depth interviews (IDIs) with 18 boys and 17 adolescent girls aged 1019years old. In total we spoke to 239 adolescents, 130 girls and 109 boys who were independently recruited for the study by Palestinian, Gaza-based, fieldworkers speaking Arabic.

### Study population and sampling approach

A purposive snowballing sampling technique was used to ensure a mix of participants from different socioeconomic backgrounds, including school dropouts, adolescent mothers, child brides, divorced/separated adolescents, and adolescents with disabilities. While we included participants benefiting from adolescent empowerment programs, we also specifically targeted particularly vulnerable adolescents, including married adolescents, their peers and families. For more information about the adolescent participants of this study, see Tables1, 2 and 3.

**Table 1:** Overview of research methods used

Method	Characteristics of participants
Focus group discussion	10 FDGs, 97 participants (39 male, 58 female)
In-depth interview	35 IDIs, 35 participants (18 male, 17 female)
Adolescent survey (QuickTapSurvey)	107 surveys, 107 participants (52 male, 55 female)
Participatory action research project	12 boys and 23 girls, aged 1519years, mean age was 16.5years, 73% are refugees.

**Table 2:** Demographic characteristics of the adolescents involved in FDGs and IDIs (*N*=132)

Variable	Percentage/number	Variable	Percentage/number
Refugees	61%	Out of school	36%
Aged 1014	39%	Male-headed household	72%
Aged 1519	44%	Having disability	17%
> 19years	17%	Social assistance beneficiary	64%
Female	57%	Adolescent services recipient	53%
Male	43%	Single	86%
Median age	15years	Married	10%
Family size 79	49%	Divorced	2%
Family size >9	34%	Separated	2%
Family income 5011000 ILS p/m	42%	No children	31%
Family income <500 ILS p/m	28%	One child	54%
Family income 1001>ILS p/m	30%	Two children	15%
In school	64%	Disability in the family	42%

**Table 3:** Characteristics of the adolescents who completed a tablet-based survey (*N*=107)

Variable	Percentage/number	Variable	Percentage/number
Jabalia camp	52 (49%)	Median age	16years
Shajaia	55 (51%)	Living in nuclear family	82 (77%)
Female	55 (51%)	Living in extended family	25 (23%)
Male	49 (52%)	In school	56 (52%)
Refugee	56 (52%)	Out of school	51 (48%)
Non-refugee	51 (48%)	Median number of siblings	6
1014years old	34 (32%)	Older than 17years old	32 (30%)
1517years old	41 (38%)		

### Study sites

The study was conducted in three diverse localities across the Gaza Strip: Jabalia refugee camp, Shajaia and Khanyounis. Jabalia camp is the closest camp to the Erez border crossing with Israel. It is home to nearly 110,000 registered refugees and is, according to the United Nations Office for the Coordination of Humanitarian Affairs (UN OCHA), home to the second largest population in severe humanitarian need [23]. Shajaia neighbourhood, in central Gaza, has around 120,000 residents, and the highest concentration of people in need [23]. It was heavily affected during the 2014 GazaIsrael war. Khanyounis, in southern Gaza, is the poorest of all Gazas governorates [24] and hosts a large refugee camp (with more than 370,000 people). As part of a participatory research pilot informing the later mixed-method research, GAGE targeted adolescents participating in empowerment initiatives undertaken by the CFTA in marginalized neighbourhoods of Khanyounis.

### Data collection and scientific rigor

A team of five local researchers (three males and two females) collected the data in Palestine. The GAGE international team based in London provided remote technical guidance and support. Pilot interviews were administered and resulted in refinement of the study tools. Local researchers used Arabic versions of the tools which have been professionally translated, revised and then tested and adapted. To ensure scientific rigour of the data, two training sessions were provided for the research team to review, understand and finalise the study tools and guidelines. To ensure credibility of data, the research team implemented a standardized approach to selecting and interviewing participants across both the quantitative and the qualitative work. Participants survey responses were checked daily for accuracy and verified through quality control measures, whilst the research supervisor listened to a subset of interview recordings from each team member to ensure the tools were being consistently applied. To facilitate triangulation of information, the relatively large sample size (for qualitative interviews) and range of respondents interviewed, using a variety of different research tools and approaches, was sufficient to obtain in-depth and triangulated information about the research questions.

### Analysis

Analysis of the quantitative data was conducted using the Statistical Package for Social Sciences (SPSS) 25 software. Descriptive analysis was followed by cross-tabulation of the variables of concern. Regarding qualitative data, as well as taking written notes, all interviews were audio-recorded. Together, these enabled the research team to prepare debriefing notes in English and check them for accuracy. Upon the completion of primary data collection at each stage, the research team took part in a two-day debriefing meeting to conduct preliminary analysis of the fieldwork findings. Qualitative interviews were then transcribed, reviewed and thematically coded. We coded the transcripts using a thematic coding structure that was informed by the conceptual framework of the GAGE programme [25]. Findings were aggregated first by instrument and then collectively across all instruments. Coding was completed using MAXQDA 12 software by a small team of experienced coders who received tailored training. The following codes were employed; availability of services, agency, quality of health services, knowledge on health, access to health information, health challenges,andperceptions on physical health. Results were compared by age, gender and location and vulnerability criteria.

### Research ethics

The research team adhered to stringent ethical measures to ensure the protection of all participants, and in particular the participating adolescents and their families, as set out under the GAGE Institutional Ethics approval document and GAGE child protection guidelines. They also followed the Modified International Code of Ethics Principles (1975) known as the Declaration of Helsinki and permission was sought from, and given by, Gazas Helsinki Committee. Participant anonymity and confidentiality were ensured, and data were securely stored. Informed consent was obtained from all participants over the age of 18years old, including from parents of underaged participants, and informed assent was obtained from adolescents aged 17 and under, prior to commencing data collection. The research lead provided a full explanation of the study and emphasised the voluntary, confidential and anonymous nature of participation.

## Results and discussion

We now turn to discuss the key findings, beginning with a review of the gendered health vulnerabilities facing adolescent girls and boys, then moving on to discuss their experiences of service provision and uptake. As Pincock and Jones note, it is critical to elicit adolescent voices and explore their lived experiences through direct engagement with them in order to set adolescent sensitive policies and programmes [26]. In humanitarian contexts like Gaza, adolescent perspectives and voices are usually unheard at various levels [13], although considering their opinions is critical as it challenges the power dynamics that marginalize young people and thus inhibit them from sharing their perspectives [26]. Malhotra et al. report [27] that it is critical to recognize that gender is at the centre of social norms that shape adolescent sexual and reproductive health outcomes, and that these norms are underpinned by power inequalities. Our findings mirror this and showcase that both age and gender have a significant impact on adolescents access to and uptake of health care services.

### Health awareness and gender norms

Adolescents in Gaza face a broad spectrum of health problems that are provoked by the protracted conflict in Gaza, with 14years of blockade and economic hardship (13, 22). Critically, as their risks are high, our findings indicate that adolescents in Gaza have limited access to information about health or healthy lifestyles. The literature shows that for boys, their main source of knowledge was friends (50%), followed by books (31%) and teachers (14%) [10]. The same source indicates that none of the participants mentioned health care providers as a source of information [10]. School health programs do exist, but these are limited in scope and often target children younger than 10years and mostly focus on discovering physical illnesses and disabilities like visual impairments [12]. Participants in this study reported that in many schools, sports classes for girls are either cancelled or replaced with regular classes; girls involvement is often not encouraged, with some girls reportedly even asked to clean classrooms instead of doing sporting activities. As one girl explained: *During sport class, we do not play or practice. Instead, we are handed sweepers to clean the place* (FGD with 1519-year-old girls, Shajaia). As Sarsour et al. noted, half of the Palestinian adolescents surveyed in Gaza were practicing a sedentary lifestyle with girls statistically being significantly more sedentary than boys [28]. Our findings also indicate that gendered social norms play a critical role in hindering girls ability to practice a healthy lifestyle. Unlike boys, during their adolescence girls are often not allowed to leave the house to do sports at gyms, as their movement outside the house is highly constrained and scrutinized. Deeply rooted social norms about protecting family honour are the main driver limiting girls movement outside the house. One 17-year-old girl from Khanyounis noted: *Many girls in Gaza become TV addicts to kill time we dont have access to other recreational resources such as girls clubs or libraries.* Another 17-year-old girl from Jabalia said: *There are no places for us. There is no cafes or sport clubs for females. Everywhere you find only men*. Our findings resonate with the literature which indicates that only 7.4% of youth in Gaza (aged 1529years) reported being a member of a sports club with a significant gender imbalance: 13.8% boys and 0.8% girls [15]. This means that most adolescent girls spend much of their time at home watching TV, vicariously observing lives they are prohibited from living. This has a negative health impact, more than half of adolescent girls in Gaza are overweight or obese [29].

In terms of sexual and reproductive health, our findings underscore that in Gaza, menstruation and puberty issues are not openly discussed due to cultural taboos. Indeed, most girls said they only approached their mothers or older sisters upon reaching menarche. This is congruent with the literature, which indicates that mothers are the main source of information about puberty for girls (70%), while friends are the main source for boys (50%) [10, 22]. Many girls reported feeling fearful and shocked by it, though the situation was harder for younger girls (aged 10 or 11) and those who had never heard about periods either at home or at school. One girl in an FGD with girls aged 1519 in Jabalia noted:

*I was 11 years old when I had my first period, I was so scared. It happened that I was doing exams. I rushed to my mother and sisters. I was scared and young. I cried for a week. I didnt know that there is something called a period, and that it happens every month.*

Given the prevailing stigma around menstruation, family members are either not aware or not supportive of girls during menstruation. In Palestine, sexual and reproductive health and menarche remains a controversial topic which is circumscribed by political, economic, cultural and religious factors [10]. Indeed, adolescent boys did not mention anything about girls menstruation, girls themselves mostly reported experiencing embarrassing comments or teasing from family members, as the following quotes illustrate:

*In Ramadan [the fasting month in Islam], when I have my period, she [younger sister] tells everybody at home and they laugh at me because I cant fast like them.* (FGD with 1519 years old, Shajaia)

*If I have my period, my husbands family gets angry and says, She has her period, that means she is not pregnant!* (FGD with 1519-year-old girls, Jabalia)

Whilst education on sexual and reproductive health, including menstruation, has been incorporated into the school curriculum in Gaza [19] and schools (teachers or counsellors) say they start introducing information about menses for seventh grade students (aged 13years), adolescent participants in this study reported that the knowledge they receive is not sufficient. Indeed, similar to the findings of other studies [19], most adolescent girls in this study say that they feel unprepared for puberty and the physical emotional changes it brings and many reported not knowing what to do when getting their first period. In qualitative research activities with adolescents, it became clear that teachers refused to talk about menses with their students, believing that mothers should discuss the topic with their daughters at home instead. One girl explained how:

*Our teacher told us to go home and ask our mothers to explain this lesson for us The teacher said let your family explain this disgusting topic!* (FGD with 1519-year-old girls, Shajaia)

Another participant reported:

*There are some teachers who would be shy and dont talk about such topics [puberty and menses] and they would skip the pages that have this in the notebook, asking us to read it alone.*(FGD with 1519-year-old girls, Shajaia).

Similarly, in Jabalia, teachers either discussed the topic superficially or provided wrong information, as one girl reported:

*In that lesson, our teacher explained that girls will have their period one day, and that means they will bleed for a week or so. This blood is poisonous, and it causes pain. Thats all she said.*(FGD with 1519-year-old girls, Jabalia).

The girl continued: *We were young, so we needed more information* (FGD with 1519-year-old girls, Jabalia).

School-based programmes represent an important missed opportunity to positively influence a range of adolescent health issues including sexual and reproductive health and have been found to increase knowledge and shift attitudes of adolescents [30]. A recent systematic review [31] concluded that when programmes worked with sectors beyond health, particularly the education sector, included multiple stakeholders, implemented diversified strategies, and fostered critical awareness and participation among community members, there was evidence for a wider systematic change.

Another highly gendered aspect of adolescent health vulnerabilities is substance abuse. Many young people in Gazaespecially adolescent malesare addicted to Tramadol, an opioid painkiller, which has also been reported to affect large proportion of the population [32]. Of Gazan young people aged 1529, 53% of girls/young women and 56% of boys/young men report that drug addiction and unhealthy lifestyles are their largest health challenge [15]. Smoking prevalence among youth has increased in recent years, up to 23.5% (40.9% of males, 5.4% of females) [16] probably due to social norms and increasing levels of stress on daily basis. Increasing access to harmful substances is exacerbated by a lack of outreach or educational programmes and a lack of integrated health and rehabilitation services [20]. Our findings suggest that substance abuse is widespread among adolescents, especially boys/young men, reflecting the many stressors they face, including unemployment and anxiety. This would reflect global trends on adolescent use of tramadol [33]. One boy commented:

*Tramadol use is common among youth. The worry comes not only from using it, but many are involved in dealing, because of the unemployment.* (FGD, older boys 1519 years old, Jabalia)

Many participants who were interviewed individually admitted having tried Tramadol or knowing people who take it. One 14-year-old, who is an orphan, noted:

*The day before yesterday, I was at a wedding party for my friend, and someone came and he had Tramadol, he put it in juice and distributes to all.* (IDI, boy, Shajaia)

Girls reported knowing people who take drugs, but did not admit to doing so themselves. Girls also reported feeling more insecure moving around in the community because of increasing substance abuse. As one girl explained:

*Males are becoming more dangerous in Gaza. Safety is less in Gaza with the many male Tramadol users They turn violent and tend to steal to secure the money needed to buy the stuff. Our families prevent us going outside in order to protect us from those bad people and thieves.* (FGD with 1519-year-old girls, Jabalia)

A 19-year-old young woman who was married early and later separated from her husband elaborated:

*I heard many stories of girls using Tramadol, my cousin and two girls at school [grade 9] are using it. They use it in the school toilets and one day, the cleaner caught them, and the school informed their parents.* (IDI with a 19-year-old girl, Jabalia)

A systematic review of peer-facilitated community-based interventions for adolescent health highlighted the evidence that working with peer facilitators significantly improves adolescent mental health and reduce substance use and violence [34]. Similarly, in the Palestinian context, research shows that prevention programmes related to alcohol consumption, drug use and sexual activity could benefit from targeting such connected behaviours collectively and adjusting expectations about peers behaviour may reduce engagement in risk taking [35].

### Age- and gender-responsive gaps in service provision

Our research highlights the range of barriers that prevent vulnerable adolescents accessing health care services in Gaza, and in particular the dearth of age-tailored information and services that respond to adolescents changing bodies and needs and resonate with the local literature [10]. For example, adolescents who did not seek treatment cited the following reasons: not knowing where to go (11%); not being able to get permission (17%); not being able to get money (36%); not being willing to go alone (particularly girls) (39%); and a lack of female health workers (32%) [10].

In terms of the specific barriers to health services uptake, a range of interconnected challenges emerged. As a boy, participant of a FGD of 1014-year-old boys in Shajaia emphasized:

*Medicines are not available*. *Doctors dont seem to be interested in treating us. They prescribe the medicines so quickly without diagnosing us well. The clinic isnt clean either.*

Whilst availability and accessibility of delivery services in Gaza are reasonable, the quality of care is often sub-optimal. Shortcomings during delivery include routine unnecessary interventions, frequent examinations, lack of privacy, lack of respect, overcrowded delivery areas, and overstretched obstetricians (who often practise in more than one institution) [10]. Adolescents also pointed out that overcrowding and uncleanliness of public health services as well as inadequate privacy act as further barriers to approaching these services.

When asked whether young people in their community ever speak to doctors or nurses about concerns, they may have about their growing bodies and puberty, only 22% said yes, while less than 5% reported having already spoken to a health care provider about such concerns. This reflects inadequate access to and utilization of adolescent-related services and information.

Adolescents also reported that medical staff at public services are often insensitive to their needs:

*In the hospital, for example, the treatment is not good unless you know someone there.* (FGD with 1519-year-old girls, Shajaia).

*As for the cleanliness of places, bathrooms there are super dirty.* (FGD with 1519-year-old girls, Shajaia).

Another girl explained that:

*I was so afraid when I went to the dentist in a public clinic. The dentist shouted at me and said if you dont want to be cured, go home! Then I went home without getting my teeth checked.* (FGD with 1014-year-old girls, Jabalia).

A 17-year-old boy noted that:

*The doctors are not good. I once went to a clinic and I was complaining of a headache and the doctor prescribed me something for my leg!!* (FGD with 1519-year-old boys, Jabalia).

Likely reflecting the stresses induced by the context of a protracted conflict, nearly half of adolescents in our tablet-based survey (53%) reported that adolescents go to see a counsellor or therapist when they are worried or sad. Of those, 16.3% indicated that they had already approached a counsellor or therapist about how they are feeling (23% boys and 9% girls). Highly gendered cultural norms shape uptake. Peer pressure, stigma related to mental health services and norms around masculinity all restrict older boys participation in counselling or psychosocial activities, which they tend to perceive as being targeted towards younger children, while regarding themselves as men. One 17-year-old boy said: *I personally feel that going to the counsellor is not good, something people who are abnormal would do* (IDI, Jabalia). On the other hand, many adolescent girls noted that they did not know about the existence of counselling and psychosocial services in the Gaza Strip. One girl lamented the lack of support for adolescents, saying: *We do not have psychosocial services here* (FGD with 1519-year-old girls, Shajaia). Organisational, cultural and psychological barriers often prevent adolescent girls accessing psychosocial services [13]. Therefore, adolescent girls reported feeling lonely and unheard, epitomised by one girl who said: *There is no one who could understand us in this context, except God, he only could understand us, but humans could not* (FGD with 1519-year-old girls, Shajaia).

Access to sexual and reproductive health care and information appears to be particularly limited. It has detrimental effects on adolescents knowledge, attitudes and practices related to sexual and reproductive health. Most boys defined puberty simply as growing up, or as one boy noted: *It means I can marry* (IDI with 16-year-old boy, Shajaia). Other signs of puberty mentioned by boys include body hair, facial hair, their voice deepening, and *feeling more like an adult*. Some boys expressed anxiety about going through puberty and the prospect of the *scary adulthood* stage. Our discussions with adolescents confirmed that they know only a little about these topics, rendering them completely unprepared for the changes puberty brings. In some instances, these feelings reflect young mens concerns over their sexual ability or ability to father children:

*I am afraid of being infertile and not being able to have children. I heard there are men who cannot have children. Im afraid to be one of them.* (IDI, 16-year-old boy, Shajaia).

Among girls, talking about sexuality is a taboo; hence sufficient information is rarely communicated and, in many situations, avoided. It was very rare for unmarried girls to mention that they had access to such information. This is creating additional challenges for adolescents: whilst the contraceptive prevalence rate has increased slightly in the past decade, unmet needs for family planning among adolescents in Gaza remain high (17%) [10]. The lack of information about health information and information about sexual and reproductive health services is compounded by social and gendered norms. Illustrative was the fact that participants noted that parents often do not allow unmarried girls to visit a gynaecologist because they are concerned that any invasive procedure might break the hymen. One girl explained:

*Fathers will prevent girls from visiting a doctor no matter how severe the condition because they believe there is a chance that her virginity will be ruined, and as a result she will not get married.* (FGD with 1519-year-old girls, Shajaia)

Even for those girls who were about to become sexually active (because they were about to marry), information was minimal. Due to their limited access to appropriate information, even on maternity-related matters, adolescent mothers appear to have insufficient knowledge/awareness of important warning signs related to sexual and reproductive health. For example, only 16% of women identified lack of foetal movement as a danger sign for their pregnancy. Only 15% of mothers were able to name at least five such danger signs (unprompted) [21]. Our discussions with adolescents confirmed that they know only a little about these topics, rendering them completely unprepared for the changes brought by puberty, marriage and motherhood. Poor communication between adolescent mothers and service providers was also evident. As an 18-year-old married girl with two infants from Khanyounis reported:

*The health personnel at the hospital were not supportive I had no information. I am a child. I dont know about these things.*

In sum, adolescents level of trust in and satisfaction with health care services is low, and this is particularly the case with married girls. Some participants described their first day of marriage as the worst experience of their life, as they were completely unaware that they would be expected to have sexual intercourse with their husband. As one girl reported:

*I had no idea what marriage was. I thought that marriage is all about supporting my husband. I had no idea that it included a sexual relationship. The biggest shock I had about getting married was at the night of my wedding, I ran away from home and went back to my family. I was terrified. My husband came to my familys home and he told them to leave me as I wish, I returned to my husband after a month, I was afraid.* (IDI, 16-year-old girl, Shajaia)

A 14-year-old married girl from Khanyounis similarly noted:

*Early marriage is a disaster. For a 14-year-old girl it is suffocation No one should be terrified like I was.*

Unsurprisingly, girls rarely talk about their sexual needs, as one girl who was married early noted:

*I do love him, but I dont feel pleased like he may do, we never talked about such things.* (IDI, 16-year-old girl, Shajaia)

Moreover, issues around reproductive health create considerable stress for girls, who rarely have a say about the timing or spacing of pregnancies, how many children they have, or what type of family planning method they use. Social, cultural, political, economic and legal constraints mean that women and girls face particular challenges in realising their full sexual and reproductive rights. For instance, a woman is required to have her partners consent to use family planning services at facilities providing family planning servicesin Gaza [10]. To a large extent, social norms also dictate that it is not acceptable for a woman to leave an infertile husband, although it is acceptable for a man to leave an infertile wife or marry another woman. As one girl explained:

*Husbands divorce their infertile wives while wives stay with them forever even if they are infertile. These women endure all difficulties and remain patient.* (FGD 1519-year-old girls, Jabalia).

The literature advocates for health services that go beyond biomedical approaches and consider the social determinants of health and address health needs holistically [13]. This can include approaches to tackle discriminatory gendered norms and barriers to service access, such as tailoring existing services to ensure gender and age-sensitivity; investing in capacity building of service providers to promote service uptake; and enhancing coordination strategies among service providers [27].

Adolescent girls who married early face particular challenges with health care services. As noted earlier, though access to antenatal care is nearly universal, the quality of services is suboptimal [10].

Participants reported concerns in prenatal care that included lack of cleanliness and privacy, waiting times and drug shortages. As one older girl commented:

*When I go to the midwife, I feel like Im in a shop not in a clinic, because everybody enters and leaves at the same time. There is no respect for appointments.* (FGD with 15-19 years old girls, Jabalia).

The gender of the health care provider can be a barrier, with most participants preferring female health care staff given the very personal nature of their problems. As one participant explained:

*I feel shy to tell a male physician that I have a severe inflammation.* (FGD with 1519-year-old girls, Jabalia).

*There are no clinics to support girls at our age staff in clinics do not understand our needs.* (FGD with 1519-year-old girls, Shajaia).

As a 17-year-old married girl from Khanyounis explained. Married at the age of 14 to a man more than twice her age, she has already been pregnant four times and has two children. Because her husband insists on many children, especially boys, against her desire. She found her first pregnancy terrifying:

*I was afraid when the baby was moving because I had never been told what to expect during pregnancy, even by doctors at the clinic.*. During delivery at the hospital, no one told her anything and *the health personnel were not supportive.*

Other participants also considered service provision by male doctors or nurses problematic, especially for unmarried girls. This was reflected in an UNFPA study too; 32% of adolescent girls surveyed reported that not having access to a female health care provider was enough of a barrier for them and resulted in them not seeking general health services [21]. Singh and colleagues note that increased service utilisation of sexual and reproductive health services by women in conflict affected settings was increased when peer-led and interpersonal education, community-based programming involving female health providers were applied [36]. In terms of sexual and reproductive health services, Jennings and colleagues noted that adolescent specific services could be included in broader sexual and reproductive health programmes, and that implementers should include adolescent people-specific strategies, targeted at both girls/women and boys/men where appropriate. The same study concludes that strategies to increase services utilisation also could include adolescent-friendly spaces, peer workers, school-based activities, always involving young people in the design of the activities that aim to improve their lives [7].

### Health service affordability

Adaptive and adequately resourced health systems are necessary to achieve good health outcomes for adolescents in conflict affected settings [13]. However, despite their families having medical insurance, most adolescent and adult study participants cited drug shortages, cost of treatment and availability of laboratory tests as among the main challenges adolescents face when visiting health facilities. Some families borrow or seek help from non-governmental organizations (NGOs) or charitable bodies to pay for medications while others either just skip treatment or use traditional remedies, explain adolescents involved in the study. This is especially true for more costly treatments, as one younger girl explained:

*I have some problems related to growing normally; and my family cant afford growth hormone education, which costs around $1,000 monthly. We take financial aid from the Ministry of Social Development and we have to borrow the rest of the money from people* (FGD with 1014-year-old girls, Jabalia).

Some girls also reported that young people try to simply endure sickness until they feel better. One younger boy explained: *When I need medicine that I cannot afford, I just sleep it off till I feel better* (FGD with 1014-year-old boys, Shajaia). In the same focus group, another boy noted that: *When I need medicine that I cannot afford, I eat garlic.* Another explained: *I do not go anywhere. I drink juice when I am sick.* And a 16-year-old adolescent mother noted a similar approach was supported by her parent:

*Once l had a stomach ache and I told my mother about it. She advised me that I have to drink boiled parsley and eat watermelon.* (IDI, 16-year-old, Jabalia)

Some girls believe that poor families would prefer to spend money on their sons because according to gender social norms and gendered opportunities for education and employment it is they who are likely to financially support the family in future, so investment in girls health is therefore a lower priority. However, adolescents generally reported that families decided on health expenditure based on the severity of the childs illness, regardless of gender. One girl commented:

*Females are usually denied health services because they dont work, while males work and earn money.* (FGD with 1519-year-old girls, Shajaia).

Boys mentioned that younger children are prioritized, followed by girls:

*Younger children go to the health facilities more because they get sick more than older ones and girls also go more.* (FGD with 1014-year-old boys, Shajaia).

Disparities were also highlighted by older girls, who thought that their parents cared more about their younger sisters health, as child illnesses are perceived as more dangerous.

## Conclusions

Unlike other studies which mainly focus on the perspectives of service providers, this article explores the lived experiences of vulnerable adolescents themselves and tells their untold stories which is vital if the 2030 Agenda for Sustainable Developments call to leave no one behind is to be realized. Additionally, it is in line with Inter-Agency Standing Committee (IASC) Grand Bargain commitments, particularly those linked to the Participation Revolution which recognises that humanitarian actors should be consulted and included.

Overall, our findings underscore the complex and interlinked challenges facing adolescents in realizing their well-being and health needs in conflict-affected contexts, and the critical role that context-specific gendered norms and practices play in shaping adolescents physical and mental well-being. Our study confirms that although adolescents basic health outcomes in Gaza are relatively good, the most pressing issues they face are related to sexual and reproductive health and risky behaviours such as smoking and substance abuse, in addition to psychosocial challenges. Limited health information can increase risky behaviours, negatively affects seeking heath care which may endanger life, and increases psychological suffering, particularly amongst girls who get married early and their children.

Our findings also show that health services are rarely tailored to the specific needs of adolescent girls and boys of different ages, which has a negative impact on service uptake. For instance, other than seeking maternity-related care or seeking treatment for acute illnesses, adolescents utilization of services is very limited due to lack of age-appropriate and gender-sensitive information and services. Even services that do not require intensive resources like counselling and health education are seldom provided. It is therefore imperative to bridge the gaps in quality of services identified both in our research and the broader literature by introducing a package of health services that are sensitive to the needs of different groups of adolescents particularly providing counselling and health education services. Adolescents involvement in the design and implementation of the services targeting them is crucial, as their involvement will critically promote their uptake of these services.

The basic package of health services in Gaza is excessively curative and does not include preventive services for adolescents, tailored to their distinct needs, especially around information and awareness. Stakeholders should therefore focus on health promotion and policy formulation to address the key health issues, including sexual and reproductive health, facing adolescent girls and boys. The provision of sexual and reproductive health information and services, particularly around menstruation, has many gaps that should be urgently addressed to reduce girls anxieties and discomfort around these issues. Significant proportions of girls in Gaza attain menarche without any understanding of what is happening to them or how to manage it. A key challenge for policy-makers and programme implementers is to intensify efforts to better inform and support adolescent girls through adolescence, from pre-adolescence to early adulthood.

Stakeholders should provide more adolescent-centred services, promote positive interactions between staff and service users, create a more adolescent-friendly environment at health facilities, and provide sufficient resources (especially educational materials, medications and menstrual hygiene management supplies). Health care providers should be supported to develop more positive and compassionate attitudes towards adolescents, focusing on caring rather than simply curing.

As many of the challenges facing adolescents are multi-faceted, it is essential to increase coordination among providers and sectors to ensure the provision of integrated health services that see health as a social rather than principally medical concept. For instance, there is a need for multi-sectoral policies and programs to promote healthy lifestyles, encourage exercise, and control obesity, drug abuse and smoking. Using mass media and social media, as well as engaging community and religious leaders, schoolteachers and other influencers, could contribute to changing norms and lifestyles. Investments in scaling up the school health programme implemented by Ministry of Health, Ministry of Education andThe United Nations Relief andWorks Agency for Palestine Refugees in the Near East (UNRWA) represents a golden opportunity to address many health-related challenges facing adolescent boys and girls. Also, using more adolescents-oriented approaches for health education such as peer to peer education, using social media and information technology is worthwhile considering. Collaborative efforts should aim to break through some of the taboos and stigma around discussing sexual and reproductive health issues, including menstruation. More efforts are also needed to collect data and evidence about the health needs of different groups of vulnerable adolescents, especially girls and boys from the most disadvantaged groups and girls who marry early.

In protracted humanitarian contexts like in Gaza, the socioeconomic determinants of health are pronounced, and young people face compounded health vulnerabilities originating from the ongoing conflict, long-standing poverty, and the limited capacity of the health system to respond to the needs of the population, particularly adolescents. Moreover, our findings underscore that in such contexts the already existing culturally rooted age and gender-based inequalities and discrimination become reinforced, especially for adolescent girls. In conflict-affected settings, the Gaza case highlights that due to ongoing hostilities and lack of adequate resources, the focus of the health system tends to be reactive and concerned with emergencies and physical injuries, with little investments in the broader vision needed to realize the SDG 3 commitment to ensure healthy lives and promote well-being for all at all ages. Health policy makers do not enjoy the minimum level of certainty to establish a coherent health system in which polices, regulations, programs and services are consistent and coordinated to address the multi-dimensional needs of adolescents especially girls in an effective way.

By focusing on the health status of adolescent girls and boys in a protracted conflict-affected context, and their experiences and perceptions of health services, this article contributes to the as yet limited international evidence. It provides insights into adolescent health status and experiences in areas characterized by intersecting, long-term compounded vulnerabilities resulting from protracted crisis and political turbulence, economic hardship, restrictive gender and age norms, weak services provision and inadequate governance which combined have exacerbate adolescents health vulnerabilities, with girls being more disadvantaged.

## Data Availability

The datasets generated and used during the current study are available from the corresponding author on a reasonable request. However, there are policies at the GAGE programme that control data sharing and exchange.

## Abbreviations

CFTA: Culture and Free Thought Association
FGDs: Focus Group Discussions
GAGE: Gender and Adolescence: Global Evidence
HIV: Human Immunodeficiency Virus
IDIs: In-depth Interviews
IASC: Inter-agency Standing Committee
NGOs: Non-governmental Organizations
PCBS: Palestinian Central Bureau of Statistics
SDG: Sustainable Development Goal
SPSS: Statistical Package for Social Sciences
STIs: Sexually Transmitted Infections
UN OCHA: United Nations Office for the Coordination of Humanitarian Affairs
UNFPA: United Nations Population Fund
UNRWA: United Nations Relief and Works Agency for Palestine Refugees in the Near East
